# An Explainable AI-Enabled Framework for Interpreting Pulmonary Diseases from Chest Radiographs

**DOI:** 10.3390/cancers15010314

**Published:** 2023-01-03

**Authors:** Zubaira Naz, Muhammad Usman Ghani Khan, Tanzila Saba, Amjad Rehman, Haitham Nobanee, Saeed Ali Bahaj

**Affiliations:** 1 Department of Computer Science, University of Engineering and Technology Lahore, Lahore 54890, Pakistan; 2 Artificial Intelligence & Data Analytics Lab, CCIS, Prince Sultan University, Riyadh 11586, Saudi Arabia; 3 College of Business, Abu Dhabi University, Abu Dhabi 59911, United Arab Emirates; 4 Oxford Center for Islamic Studies, University of Oxford, Oxford OX3 0EE, UK; 5 Faculty of Humanities & Social Sciences, University of Liverpool, Liverpool L69 7WZ, UK; 6 MIS Department, College of Business Administration, Prince Sattam bin Abdulaziz University, Alkharj 11942, Saudi Arabia

**Keywords:** explainable AI, class activation map, Grad-CAM, LIME, coronavirus disease, reverse transcription polymerase chain reaction, computed tomography, healthcare, health risks

## Abstract

**Simple Summary:**

Different chest diseases badly affect the human respiration system. The chest radiographs of the lungs are used to classify these diseases. Identifying diseases is essential, but the most important thing is explaining the reason behind classification results. This research provides an explanation of the classification results of different lung pulmonary diseases so that doctors can understand the reason that causes these diseases. This work achieved 97% classification accuracy. This research also evaluated the highlighted regions in the input image, during the explanation of classification results with the manifest file, where the doctor highlighted the same regions with red arrows. The automatic disease explanation and identification will help doctors to diagnose these diseases at a very early stage.

**Abstract:**

Explainable Artificial Intelligence is a key component of artificially intelligent systems that aim to explain the classification results. The classification results explanation is essential for automatic disease diagnosis in healthcare. The human respiration system is badly affected by different chest pulmonary diseases. Automatic classification and explanation can be used to detect these lung diseases. In this paper, we introduced a CNN-based transfer learning-based approach for automatically explaining pulmonary diseases, i.e., edema, tuberculosis, nodules, and pneumonia from chest radiographs. Among these pulmonary diseases, pneumonia, which COVID-19 causes, is deadly; therefore, radiographs of COVID-19 are used for the explanation task. We used the ResNet50 neural network and trained the network on extensive training with the COVID-CT dataset and the COVIDNet dataset. The interpretable model LIME is used for the explanation of classification results. Lime highlights the input image’s important features for generating the classification result. We evaluated the explanation using radiologists’ highlighted images and identified that our model highlights and explains the same regions. We achieved improved classification results with our fine-tuned model with an accuracy of 93% and 97%, respectively. The analysis of our results indicates that this research not only improves the classification results but also provides an explanation of pulmonary diseases with advanced deep-learning methods. This research would assist radiologists with automatic disease detection and explanations, which are used to make clinical decisions and assist in diagnosing and treating pulmonary diseases in the early stage.

## 1. Introduction

The human respiratory system provides respiration using the lungs, which are fundamental organs of the human body. Lung pathology is observed through chest radiographs, known as chest X-rays (CXRs) [1]. Many pulmonary diseases are diagnosed by observing different pathological patterns through CXRs [2]. Computed tomography (CT) and CXR are low-cost and effective techniques for detecting pulmonary diseases such as tuberculosis, edema, nodules, and pneumonia [3]. Among all these pathologies, pneumonia is a fatal one that is clinically measured by observing lobar consolidation, interstitial opacities, and airspace opacities. Edema is identified by pulmonary vessels, patchy shadowing, increased cardiac size, and septal lines [4]. The CXRs are also used to identify tuberculosis by observing the cavities and consolidations in the upper zone of the lungs.

On the other hand, the nodules are identified as a spot in the lung zones using CXRs [5]. In the past years, there was an unexpected rise in COVID-19 patients who also had deadly lung infections such as pneumonia [6]. COVID-19 is identified by observing the airspace opacities, lobar consolidation, and patchy shadow [7,8]. This primarily affects the pulmonary system, causing a chronic inflammation that severely lowers overall lung capacity [9]. This is a severe and deadly disease due to its high transmission, absence of general population immunity, and long incubation period. CT and CXR are the primary imaging diagnostics for these pulmonary diseases [10].

This manual diagnosis process takes more time which was the main concern. Therefore, deep learning (DL)-based approaches are being employed for automated pulmonary lung disease identification [11] to deliver accurate results. DL produces highly detailed images and CT scans, the standard method for lung diagnosis and treatment [12,13]. However, it is still being determined how these DL algorithms reach the classification results and which features are more important to produce that output [14,15]. This shows deep learning algorithms’ inherent black-box character and other factors, such as processing costs [16]. It originates from the inability to represent the information for a given job completed by a deep neural network, despite understanding the basic statistical principles. Easier artificial intelligence (AI) methods, such as decision trees and linear regression, are self-explanatory since the classifier boundary can be depicted in a few dimensions using the model’s parameters. However, tasks such as the classification of 3D and most 2D medical images lack the complexity needed and lack the tools to check the behaviour of black-box models, thus having a negative impact on the deployment of deep learning in a variety of fields, including finance and automated vehicles and especially healthcare, where explainability and reliability of classification of disease are critical factors for end-user trust [17].

Explainable AI (XAI) has the key to opening the deep learning “black box” nature [18]. XAI is an AI model that explains goals, logic, and decision-making to laymen [19]. End users in this case could be AI system creators or those influenced by an AI model’s judgment [3]. The findings show that quantitative and qualitative visual representations can help clinicians understand and make better decisions by providing more detailed data from the learned XAI algorithms’ results [20]. In healthcare-related medical imaging problems, the accuracy of the prediction model is essential. Still, the visualization and localization of input medical images are more significant, which helped to identify the main regions contributing to the classification results [18]. Even though there are many reasons why XAI is substantial, research reveals that the three most critical problems are: (1) trustworthiness, (2) transparency, and (3) bias and fairness in algorithms [21]. With these features, XAI has plenty of applications in different domains for explaining deep learning algorithms’ prediction. In healthcare, XAI is important for explaining deep learning algorithms’ classification.

In this research, we introduced the concept of explainability for detecting the important features in medical images where the classification model gives extra attention throughout the classification task. We present the deep-learning-based framework for explaining pulmonary diseases using chest radiographs. All pulmonary diseases badly affect the lungs and respiration system of humans but have different affected zones. Due to a large number of cases of pulmonary disease COVID-19 in the past years, we took radiographs of COVID-19 for the classification results’ explanation task. We achieved the goal of identifying the COVID-19 disease and provided the visualisation of input medical images contributing to the classification results. First, we provided the CXR images as input to our deep-learning-based system. The system processed the input image and provided the classification result. After that, the CXR image is passed to our XAI local interpretable model agnostic explanations (LIME) to determine which specific features helped the deep convolution neural network distinguish between COVID-19 and non-COVID-19 patients. LIME provided the highlighted regions of the input CXR images. That explains the classification results’ reasons in the form of the highlighted segment of that image with different colors. In the last step, we evaluated the doctor-highlighted region with the model, and it provided the same highlighted components.

Further, this research has four main sections; Section 2 presents the in-depth state of the art; Section 3 presents the proposed methodology; Section 4 exhibits results; finally, Section 5 concludes the research.

## 2. Literature Survey

DL techniques enhanced the performance of medical imaging diagnostic frameworks, especially for abnormal pathologies and pulmonary diseases of lungs from CXRs [22]. Most of these systems used transfer learning approaches for identifying different lung pulmonary diseases using chest radiographs. These techniques are used to identify pulmonary disorders, i.e., edema, nodules tuberculosis, pneumonia, and COVID-19 through chest radiographs [23]. In medical imaging, disease identification is important, but explanation and interpretability also play an important role [24]. XAI provides the reason behind the specific classification and prediction results. XAI’s primary goal is to investigate and develop methods for explaining the individual predictions of DL systems. We understand that a clear explanation of the reached decision is critical in medical applications depending on images. In an ideal case, the system makes a decision based on the input data and justifies which image part led to a certain classification result [25]. XAI was recently considered because of its potential to provide an understanding of the behavior and process of some complex deep-learning models. Several studies [26] showed that, using a decision tree and linear models, it is easy to explain approaches in a way that is easy to comprehend and interpret for humans. In this paper, we took the case study of COVID-19 from all pulmonary diseases. The literature survey of some of the existing XAI systems for the classification of lung diseases is presented in Table 1.

Ye et al. [18] used ResNet 101 to identify lung pulmonary disease COVID-19 using CT scans. They also used the Class Activation Map (CAM) for global explanation and achieved a classification accuracy of 85%. They used the concept of explanation in the medical image classification and tried to explain the results using the XAI approach CAM. In another research project, Lucas O. Teixeira employed two XAI techniques to analyze the effect of human lung segmentation, predict lung diseases, and provide the explanation using LIME and Gradient-weighted Class Activation Mapping (Grad-CAM). LIME works by identifying features, such as superpixels (picture zones), that improve the likelihood of the expected class, i.e., areas that support the present model prediction. Since this model actively employs such regions to produce predictions, they might be considered important. Grad CAM examines the gradients that flow into the last convolution layers of a CNN for an input image and label. The activation mapping (AM) can then be examined visually to ensure that the model focuses on the correct area of the input image. They used UNET architecture to classify lung diseases, pneumonia, lung opacities, and COVID-19 and achieved an accuracy of 83% [24].

In another research project, Linda W et al. used the COVIDNet data set to train the VGG16 network for the classification of COVID-19 tasks. They used 3975 CXRs for training the model. GSInquire explains the classification task. They achieved an accuracy of 83% [27]. After some time, Lin Zou et al. explained pulmonary diseases, pneumonia and COVID-19 using chest X-rays. They used 2235 x-rays and explained using ensemble XAI with Grad-Cam++ and SHAP. They achieved 87% classification accuracy [28]. Similarly, Md. Rezaul Karim et al. [29] developed a system using DenseNet, ResNet, and VGGNet models named DeepCovidExplainer that provides the explanations of classification results of COVID-19. They used 5959 CXR from patients to classify the normal, COVID-19, and Pneumonia classes and achieved 90% classification accuracy. Literature studies of existing pulmonary lung disease identification using DL techniques are presented in Table 2. L. O. Hall et al. [31] examined the lung diseases pneumonia and COVID-19 from the chest X-rays using the latest techniques of DL. They used DL architectures (VGG-16 and Resnet-50) to classify the diseases into two categories. They used a small dataset containing 135 chest X-rays of COVID-19 and 320 chest X-rays of pneumonia. They achieved satisfying results of 82.2% even though the dataset used was limited. M.K. Pundit et al. used deep neural network architecture VGG16 on 1428 chest X-rays images. They focused on the identification of the lung disease COVID-19. They improved the accuracy by a little to 92% [32]. After the success of predicting COVID-19 from chest X-rays, M. Singh et al. applied a machine-learning-based algorithm (Support Vector Machine) to CT scan data to classify COVID-19. They used a transfer-learning-based support vector machine on VGG16 architecture. Their dataset consists of 718 CT Scan images; 349 of them are of COVID-19, and 376 are of non-COVID-19. Their results were promising as they achieved a ROC Score of 85.3 and an accuracy of 83.5% [33]. CoXNet, a multi-dilation CNN, was used for the automatic discovery of COVID-19 by T. Mahmud et al. They also worked on X-ray pictures but with convertible multi-accessible feature optimization. Their dataset consists of four classes, COVID-19, Normal, Viral, and Bacterial pneumonia. Each class has 305 X-ray images. They achieved 80.2% accuracies along with a ROC score of 82.0 [34].

Segmentation techniques were used by M. Aleem et al. [35] to fragment the symptoms of COVID-19 in the CT SCANS of the chest. The latest techniques of DL such as RCNN were used with the backbone of Resnet. The system was trained on 669 CT scans having 313 positive COVID-19 patients and 356 healthy ones. They achieved an accuracy of 83% with ROC scores of 85. With time, researchers kept working hard and coming up with new techniques as some researchers did [36] using SVM-based analysis of X-ray images. They used support vector machines to differentiate between COVID-19 and normal subjects. The dataset that was used for training and testing their system was 1380 CT scans; however, the results were not that promising, with an accuracy of 63% and the ROC score of 72. M. Pandit et al. worked on chest radiographs to detect the lung disease COVID-19. They used techniques and achieved outstanding results. VGG-16 is used for classification purposes. The dataset used for training and testing the system contains 1428 chest radiographs with bacterial pneumonia, healthy, and COVID-19. They attained an accuracy of 82.5% [37].

Xingyi Yang et al. [30] provided a COVID CT dataset verified by the senior radiologist of Tongji Hospital, Wuhan, China. They collected the data on COVID-19 and normal CT images for the diagnosis of COVID-19; they collected data from different patients and provided the manifest file of that data as well. They also developed an automatic disease detection technique using these CT images and achieved an accuracy of 85%. We will use this COVIDNet and COVID CT dataset and improve the classification results. In the medical domain, experts are required to explain the reasons for classification results manually. We are developing a framework that visually explains the deep learning classification model results. We provide an output image highlighting the important features that participate in the classification results. We evaluate the proposed model explanation with the radiologist highlighting glass ground opacities in the CXRs.

The main contributions of this research are:

- In this research, an explainable AI framework is developed for detecting pulmonary diseases where the classification model gives extra attention throughout the classification task using chest radiographs.
- For the classification task, transfer-learning-based Resnet50 architecture is used. This developed system secures superior classification accuracies compared to the existing approaches by achieving 93% and 97% of pulmonary disease COVID-19.
- Interpretable Model-agnostic Explanations (LIME) are used to explain the classification results. This unique explanation method may explain any classifier’s predictions in a comprehensible manner that provides the explanation in the form of highlighted regions in the input image in which part of the image is used in the classification result.
- For the evaluation of the explanation task, two CT images from a journal [38] are used that are diagnosed and highlighted by a verified doctor. This research paper shows that the interpretable model explains the same region that is highlighted by a doctor.

## 3. Proposed Methodology

**Figure 1:**
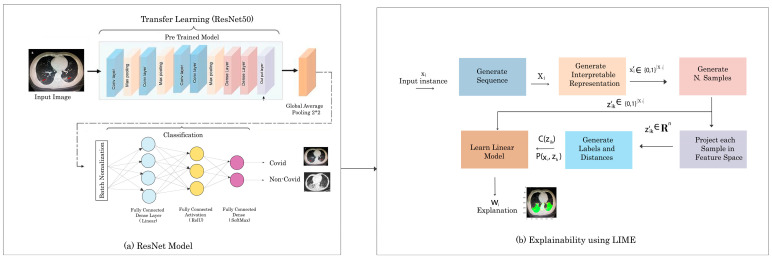
The architecture diagram of the proposed method.

The proposed methodology has a sequence of steps that include dataset understanding, in which we understand the chest radiographs of humans and the importance of various regions present in the CXRs images. The second step is feature map generation which generates the feature maps of those CXRs images. We used the concept of transfer learning in our methodology and used pre-trained Resnet50 for the classification of COVID-/NON-COVID. Our final step is to explain the pulmonary disease classification results visually using the interpretable model LIME. This developed framework takes a CXR image as input. After that, it classifies the input image as COVID and NON-COVID. Once the decision is made, we pass that image and classification prediction to the proposed explainable model LIME, and then LIME will highlight the region of the input image. That highlighted region shows which part of the CXRs images took part in the classification results. LIME highlights the important features of the image with color. We evaluate our color region with the manifest info file of the COVID-CT dataset. We check that the doctor mentioned the same region while examining the CT scan of the COVID-19 patient so our model is giving the same region. The complete workflow diagram of our methodology is shown in Figure 1.

### 3.1. Dataset

The datasets are the backbone of every proposed method and architecture in the computer vision and deep-learning domain. Any deep-learning system’s accuracy is directly proportional to the above-mentioned parameter. Therefore, the proposed model is about a vision-related problem; a dataset is required due to deep learning. We used the COVID-CT [30] dataset and COVIDNet for COVID classification, and then we explain their classification results. The dataset’s complete CXRs images and classes are given in Table 3. The COVID-CT dataset includes 349 COVID-19-positive CT scans from 216 individuals and 397 COVID-19-negative CT pictures from 397 patients. The dataset is freely available to the public in order to promote COVID-19 CT-based testing research and development. The distribution of the dataset into training, testing, and validation is shown in Table 4.

**Figure 2:**
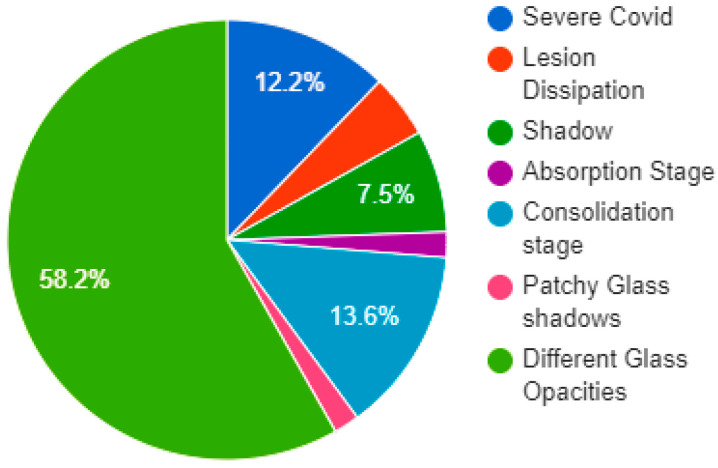
Chart of distribution of the proportion of CT manifestations of COVID-19.

This dataset has the manifest information of each image that helps the researcher understand the data images easily. In addition, the manifest file has information about the patients’ medical history and lung scans. Figure 2 represents the distribution of the proportion of CT manifestations of COVID-19. We used these mentioned features in the explainability of chest radiographs.

### 3.2. Proposed CNN Model

Convolutional neural networks (CNNs) are a type of deep neural network that is utilized in image recognition. In order for the CNN to function, the images provided as input must be recognized by computers and translated into a processable format. As a result, CXR images are transformed to matrix format first. Then, based on CXR image differences and matrices, the system identifies which image belongs to which label. During the training phase, it learns the consequences of these changes on the label and then uses them to create predictions for fresh CXR images. We used a transfer learning technique to transfer already learned weights to pass into the current deep learning problem. The parameters of our transfer learning model are learning rates of 1 × 10^−4^, 100 epochs, a batch size of 32, and two classes either “COVID” or “Non-COVID”. Due to the small capacity of the dataset in the current task, deep CNN can collect general characteristics from the source dataset using the transfer learning approach. The transfer learning algorithms have a number of benefits, including avoiding overfitting whenever the number of training samples is restricted, lowering computation power, and speeding up system convergence. Figure 1a shows the complete flow of our Res-net50 model. We utilized batch normalization for each mini-batch to standardize the inputs to a layer. It normalizes the input layer and rescales it to speed up the training process and for improving stability. Equations (1)–(4) is the mathematical representation of Batch Normalization:

(1) $$\mu =\frac{1}{n}\sum_{i}Z^{\left(i\right)}$$

(2) $$\sigma^{2}=\frac{1}{n}\underset{i}{\sum }{\left(Z^{\left(i\right)}-\mu \right)}^{2}\;$$

(3) $$Z_{\mathrm{norm}}^{\left(i\right)}=\frac{Z^{\left(i\right)}-\mu }{\sqrt{\sigma^{2}-\in }}$$

(4) $$Z={\;\gamma \;*Z}_{\mathrm{norm}\;}^{\left(i\right)}+\beta$$

Here, the mean is *μ* and the variance is σ; *ε* is a constant used for numerical stability; the activation vector is $Z^{\left(i\right)}$; *γ* allows for adjusting the standard deviation; *β* allows for adjusting the bias. Batch normalization made the training of the network faster. There are two main ways of using learning algorithms from pre-trained networks in the context of deep learning, extraction and fine-tuning of features. In our case, we used the second approach to modify and fine-tune the traditional ResNe50 architecture. That helps to outperform feature extraction and achieves better performance. The modified Resnet50 architecture generates the transfer feature map. The training of the Resnet50 model is conducted by using available CXRs data and the transfer learning pre-trained weight. For normalizing a neural network’s output to a probability distribution over expected output classes, the SoftMax function was employed as the final activation function. It converts real values into probabilities by dividing the exponential of a particular class by the sum of the exponential of all classes. In class, higher probability is considered the output prediction. Totals of 349 COVID-19-positive CT scans and 397 COVID-19-negative CT were used for training the proposed model for COVID-CT data, and 19,000 CXRs were used for the COVID-NET dataset. After the training, we then saved the trained model that will be further used in the classification task. The pseudocode for modified ResNet50 is given below.
**Pseudocode** ResNet50**Input:** Chest Radiographs **Output:** classification results: Covid Or Normal **Start**  *lr* ← 1 × 10^−4^           ▷ lr is Initial_Learning_rate  *Batch_Size* ← 32  *Number_of_Epochs* ← 28  *Base_Model* ← **ResNet50**(*weights* ← “*imagenet*”, *include_top* ← *False*,   *input_tensor* ← Input (*shape* ← (*224, 224, 3*)))  ▷ ResNet50 is the base Model   *headModel* ← baseModel.output  *headModel* ← AveragePooling2D(pool_size ← (7, 7))(headModel)   *headModel* ← Flatten(name ← “flatten”)(headModel)  *headModel* ← Dense(256, activation ← “relu”)(headModel)  *headModel* ← Dropout(0.5)(headModel)  *headModel* ← Dense(len(CLASSES), activation ← “softmax”)(headModel)  model ← Model(inputs ← baseModel.input, outputs ← headModel)  **for** layer in baseModel.layers:      layer.trainable ← True  **end for**  opt ← optimizers.Adam (lr ← *INIT_LR*, decay ← *INIT_LR*/*Number_of_Epochs*)  model.compile (loss ← “binary_crossentropy”, optimizer ← opt, metrics ← [“Accuracy”])  H ← model.fit_generator (trainGen, steps_per_epoch ← totalTrain, validation_data ← valGen, validation_steps ← totalVal, epochs ← Number_of_Epochs) **End**

### 3.3. Classification and Explanation

**Figure 3:**
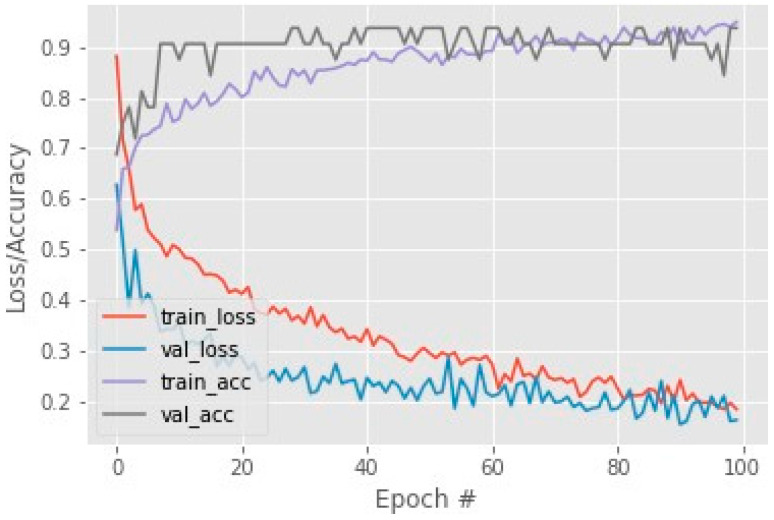
COVID-CT dataset training loss and accuracy.

Instead of presenting our own architecture, available deep CNN architectures demonstrated greater performance across a wide range of classification problems. ResNet50 has a 50-layer variation of the residual neural network. Residual networks offer excellent performance and feature count balance and a high training speed. Another advantage of the residual network architecture is that it used different sizes of images for training. ResNet50′s weights are pre-trained on the ImageNet dataset. This pre-trained model can be used to classify pulmonary diseases COVID and NON-COVID. Figure 3 shows that our system took the input CXR and provided the classification. The final step of the proposed system was to explain the DL model and the reason behind the classification results.

The LIME interpretable model is used to explain and highlight the important features that contributed to the classification result of pulmonary lung diseases. The sequence of steps of the LIME Algorithm that we used to explain our classification results is given below in Algorithm 1.
**Algorithm 1.** LIME**Require:** Classifier f, Number of samples N **Require:** Instance x, and its interpretable version $x^{\prime }$ **Require:** Similarity kernel πx, Length of explanation K Z ← {} **for** *i ∈ {1, 2, 3,..., N}* **do**  $z_{i}^{\prime }$ ← Sample around ($x^{\prime }$)  Z ← Z ∪ ($z_{i}^{\prime }$, f$(z^{i}$), $\pi_{x}$ ($z_{i}$)) **end for** *w* ← *K-Lasso(Z, K)*     ▷ with $z_{i}^{\prime }$ as features, f(*z*) as target **return** w

Figure 1b shows the steps involved in the explainability using LIME. First, LIME used CXR images as input and generated the sequence present in the image. After that, it generated the interpretable representations and generated N samples. Then, it matched each sample with the featured map of the input CXR images and calculated the predicted label and distance from the predicted output. Following, these labels and distance values were passed to a linear model that provided the explanations, and a specific result was produced. LIME also highlighted the region in the CXRs image, which showed which part of the image took part of the output. The system used a CXR image as an input and classified the image as COVID and NON-COVID, and then LIME highlighted the important regions in the image, which can clearly represent and explain the reasons for classification results.

(5) $$\underset{g\varepsilon G}{\xi (x)=\mathrm{argmin}\;}{L\;(f,\;g,\pi }_{x})+\Omega (g)\;$$

The Equation (5) is used for the LIME explainability calculation. In this equation, f is the model classifier, and *G* is a class of interpretable models. *gG* shows the learning of a local explainable model, and is x the proximity measure. (g) is used to measure the model complexity. The loss or distance function is denoted as L (f,g). After computing the explainability, evaluation is carried out using some images from verified doctors.

## 4. Results and Discussion

This section presents the complete experiment setup, performance metrics, and results of our classification deep learning models. We also discuss the results of the explain-ability model that we used and show the highlighted regions. Finally, we evaluate the explainability using our deep learning model highlighted region and compare it with the doctor-identified region.

### 4.1. Experimental Setup

In this research, we used suggested deep transfer learning models that were trained using the Python language. All experiments were run on a Google Colaboratory (COLAB) and the online cloud services with a free Central Processing Unit (CPU), NVIDIA K80 Graphics Processing Unit (GPU), and Tensor Processing Unit (TPU) hardware. By optimizing the cross-entropy value with CNN models, ResNet50 was pre-trained with some random initial weights. For overall experiments, the batch size, learning rate, and the number of epochs were set at 3, 1 × 10^5^, and 30, respectively. All samples were randomly divided into two distinct datasets, with 80 percent used for training and 20 percent used for testing. The k-fold approach was chosen as the cross-validation method, and results were obtained using five distinct k values (k = 1–5). We first performed experiments using different CNN architectures such as DenseNet169, MobileNet, COVID LargeNet, and Resnet50. We trained these models using the COVID-CT dataset and calculated the training, testing, and validation accuracies. We found that the ResNet50 performs best of all of them. We used the transfer learning concept, fine-tuned the Resnet50, and found the best possible results. The results of different CNN models are shown in Table 5.

We selected the Resnet50 model for our COVID-19 disease detection from the CXR images after performing the experiments on different CNN models as we finalized the Resnet50 model. We improved the base results; the next step was to explain the classification results. However, before moving toward the final step, we performed some more experiments and for that purpose, we trained the Resnet50 model on another dataset. The second dataset was the COVIDNet dataset which has more classes. We used that dataset for our problem and trained the Resnet50 on COVID-CT and COVIDNet datasets. We calculated the results on both datasets, shown in Table 6. For the calculation of the final results, we used some performance matrices that are discussed below.

### 4.2. Performance Matrices

In this paper, five parameters were used for measuring the performance of deep transfer learning models, having their advantages and disadvantages. We describe them one by one in the following Equations (6)–(10).

Accuracy: The correct predicted cases divided by a total number of cases gives us the accuracy [19]. High accuracy means the model is predicting accurately. It is actually a sum of true positives and negatives which is TP + TN divided by the sum of TP (True positives), TN (True Negatives), FP (False positives), and FN (False negatives).

(6) $$\mathrm{Accuracy}\;=\frac{TP+TN}{TP+TN+FP+FN}$$

Precision: Precision is called a number of the correct results out of the predicted results. It is calculated by dividing true positives by the sum of true positives and false positives.

(7) $$\mathrm{Precision}\;=\frac{TP\;}{TP+FP}$$

Specificity: A number of valid negative predictions divided by a total number of negatives is known as specificity.

(8) $$\mathrm{Specificity}\;=\frac{TN}{TN+FP}$$

Recall: The recall is defined as a number of the positive predicted results out of the total positive cases, also known as Sensitivity and termed as the true positivity rate. It is measured by true positives which are divided by the sum of true predictions.

(9) $$\mathrm{Recall}\;=\frac{TP}{TP+FN}$$

F1. Measure: The harmonic average of precision and recall is used to get the F1 score. To refresh your memory, the harmonic mean is indeed an alternative to the more commonly used arithmetic mean. When calculating an overall average, it is very useful.

F1 Measure = 2 × Precision × Recall Precision + Recall(10)

**Figure 4:**
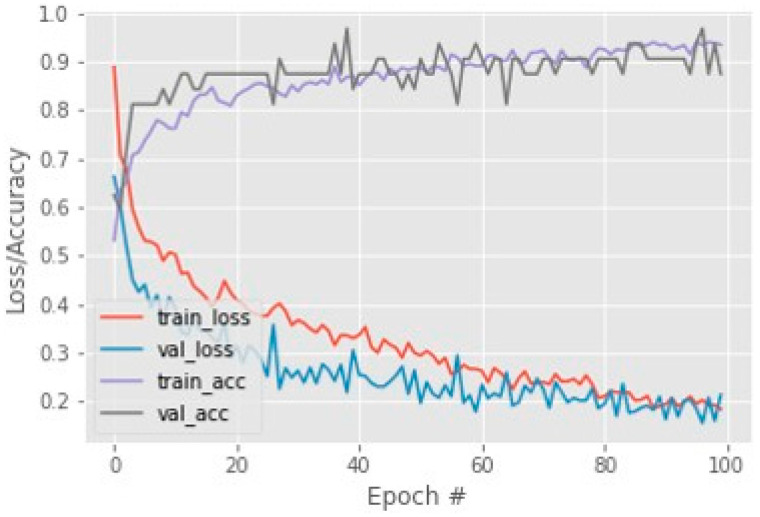
COVIDNet dataset training loss and accuracy.

By using these performance measures, the loss and accuracy of the Resnet50 model calculated and accuracy on COVID-CT testing data are shown in Figure 3. This achieved accuracy is 93% on 100 sets of epochs and for this dataset. The COVIDNet dataset has 97 accuracies on the Resnet50 model which is shown in Figure 4.

**Figure 5:**
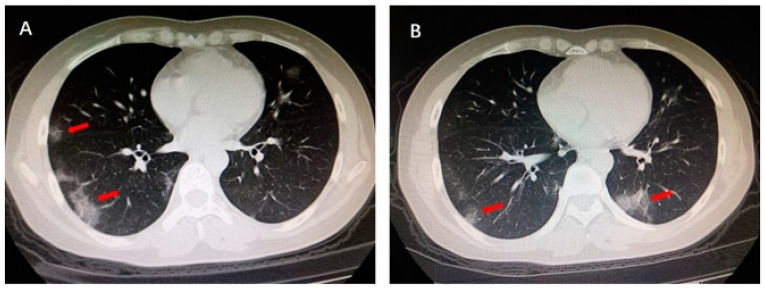
Multiple patchy glass ground opacities in bilateral subpleural areas (red arrow).

The LIME interpretable model was used for the explainability of lung pulmonary disease COVID-19. After understanding the manifest info file of the COVID-CT dataset, we found some recurrence of the positive COVID CT images that is published in the 2020 International Journal. Figure 5 shows the region highlighted by the arrow by one of the verified doctors, and he describes the reasons that caused the COVID-19. This red arrow shows the multiple patchy ground-glass opacities in bilateral subpleural areas. These are the main features of the CT images that took part in the COVID-19 classification result.

**Figure 6:**
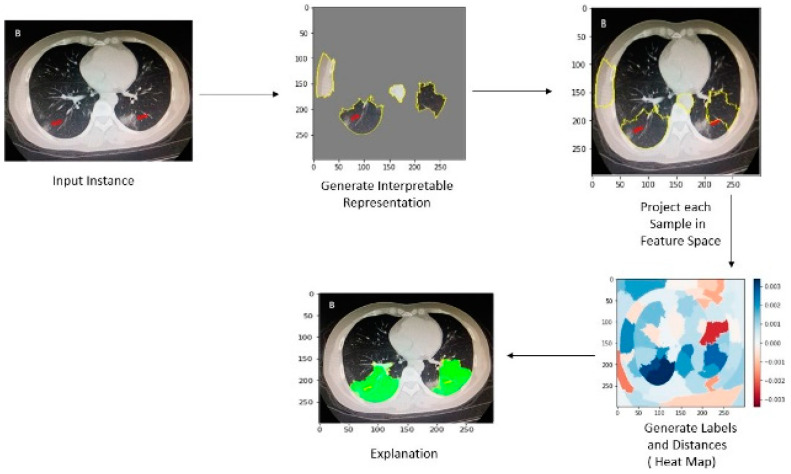
Explainability using LIME on CT-Image.

The main goal of this paper is to explain the same regions that are highlighted by the doctor after we classify the CT image as COVID. LIME took the same sample instance of the COVID-CT image and step-by-step process of the image as shown in Figure 6. First, it generates boundaries in the input image, finds the distance between the actual and predicted feature map, and generates the label. Then, it shows the distance using a heat map and highlights the region with color patches. These regions are the important feature that took part in the classification results.

**Figure 7:**
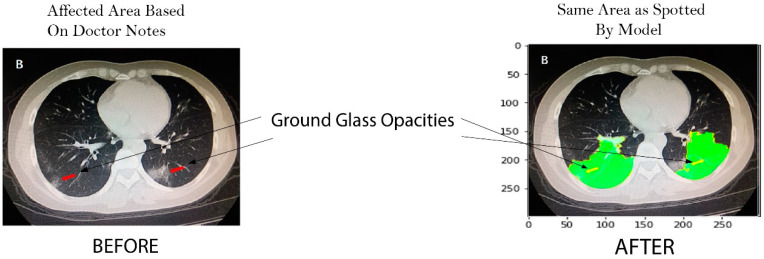
Before: Ground glass opacities highlighted by doctor, after: Same area highlighted by our model.

This research achieves the main goal using the LIME interpretable model. Further, results are evaluated using a recurrence image to verify model authenticity by cross validation of experts as shown in Figure 7.

### 4.3. Comparative Analysis

The qualitative and quantitative comparative analysis is conducted with the state-of-the-art methods. For quantitative analysis, we chose various state-of-the-art approaches for comparison that performed well on COVID-19 classification. Instead of analyzing each CXR image, we used the transfer learning approach to train the Resnet50 network on the entire CXR images. Meanwhile, COVID-CT and COVID-Net were used for the classification of COVID and NON-COVID. The results are shown in Table 6. We can view that our developed framework achieved the highest accuracy.

Along with its quantitative solid performance, the proposed model’s explainability is also promising. To enhance the prediction to be more explainable, the activation maps are extracted by the developed method’s explainable module, which we visualized in Figure 7. It can be seen that the proposed method would make a positive prediction by focusing on the most important section of the CXR image, which can be designated as the bilateral subpleural areas that show the ground glass opacities of the lungs. Furthermore, rather than focusing solely on the image’s most important regions, we also consider the local regional contribution to the forecast. As previously stated, the input images were separated into many super-pixels, each of which had a similar visual pattern. This method highlighted those super-pixels in each image that greatly contributed to the prediction, and we can see that the regions with glass opacities are clearly highlighted for such a prediction. The comparative analysis of different XAI systems’ clinical features is available in Table 7. This research selects more clinical features to be explained in the developed framework and provides greater explainability than the other available methods. In this research, the developed explainable AI framework provides an explanation of local and global features. As a result, it reveals that diseased areas can be easily identified using this system. The proposed method explains pulmonary disease identification that can be used as a valuable diagnostic tool for doctors.

## 5. Conclusions

An explainable AI-based framework was proposed in this research to address the challenge of classification result explainability in the healthcare domain using medical images CXRs. This research presented a framework that provides the explainability of lung pulmonary diseases, i.e., edema, tuberculosis, nodules, pneumonia, and COVID-19 using chest radiographs. This research used CXRs data from the two datasets COVID-CT and COVIDNet to train the transfer-learning-based Resnet50 CNN model. This developed system achieved improved classification accuracies of 93% and 97% on both datasets. After classifying the pulmonary disease, this research further explains the classification results by using the interpretable LIME model. Our developed framework explains the classification results of the input CXRs image and highlights the region of the image that participates in the classification results. These highlighted regions are the important features that are used in the classification of diseases. After that, we evaluated our explanation results by a doctor-highlighted region image from the manifest file of the COVID-CT dataset and found that our model highlights the same ground glass opacities regions as those highlighted by the doctor. Evaluation and testing show that our approach can explain the classification results using chest radiographs. This automatic classification and explanation of lung pulmonary diseases can assist radiologists to detect and diagnose deadly lung diseases at an early stage.

## Figures and Tables

**Table 1:** Comparative analysis of existing explainable artificial intelligence (XAI) and classification models for lung diseases.

Methodology	Dataset	Explainability Models	Accuracy %
ResNet 101 [18]	897 CT Scans	CAM, LIME	85%
U-Net CNN [24]	1478 X-rays	Grad-CAM, LIME	83%
VGG16, ResNet [27]	3975 CXRs	GSInquire	83%
Xception [28]	2235 chest X-rays	SHAP, Grad-CAM++	87%
DenseNet, ResNet, VGGNet [29]	5959 CXRs	Grad-CAM++, LRP	90%
DenseNet169 [30]	787 CT Scans	Not Used	85%
Proposed Mode	787 CT Scans, 10,000 CXRs Scans	LIME	93%, 97%

**Table 2:** Comparative analysis of the existing classification models for pulmonary diseases using lung scans and X-rays.

Methodology	Dataset	Accuracy %
VGG16 [31]	455 X-rays	82.2%
VGG-16 [32]	1428 X-ray	92%
VGG16, SVM [33]	718 CT scans	83.5%
CovXNets [34]	305 X-rays	80.2%
RCNN, ResNet, ResNet101 [35]	669 CT scans	83%
SVM [36]	1380 CT scans	63%
VGG-16 [37]	1428 CT scans	82.5%

**Table 3:** Total sample and classes in COVID-CT and COVID-Net datasets.

Dataset	Total	Classes
COVID-CT	800	2
COVID-NET	19,000	3

**Table 4:** Sample data distribution in test, training, and validation of both COVID and non-COVID class.

Type	Non-COVID	COVID
Train	234	191
Test	58	60
Validate	105	98

**Table 5:** Results on different CNN models.

CNN Model	Accuracy %
DenseNet169	85
MobileNet	83
COVID LargeNet	88
Our Model	93

**Table 6:** Classification results with ResNet50.

Measures	COVID-CT Dataset	COVID Net Dataset
	COVID or NON-COVID	COVID or NON-COVID
Precision	87-93	98-93
Recall	92-88	92-98
F1 Measure	90-90	95-95
Accuracy	93	97

**Table 7:** Clinical features analysis with XAI System.

XAI Methods	XAI Clinical Features	Agnostic or Specific	Global/Local
GSInquire [24]	Absorption Area	Specific	Local
SHAP, Grad-CAM++ [25]	Glass Opacities	Specific	Local
Grad-CAM, LIME [21]	Glass Opacities	Specific	Global
DeepCOVIDExplainer [29]	Lesion Dissipation	Agnostic	Local
Proposed XAI Model (LIME)	Lesion, Dissipation, Consolidation area, Absorption area, patchy Glass Shadow, Glass Opacities	Agnostic	Both

## Data Availability

Publicly available datasets were analyzed in this study. This data can be found here: http://arxiv.org/abs/2004.02060.
